# Physical Ageing of Amorphous Indapamide Characterised by Differential Scanning Calorimetry

**DOI:** 10.3390/pharmaceutics12090800

**Published:** 2020-08-25

**Authors:** Agata Drogoń, Marcin Skotnicki, Agnieszka Skotnicka, Marek Pyda

**Affiliations:** 1 Department of Chemistry, Rzeszów University of Technology, 35-959 Rzeszów, Poland; agata.drogon@yahoo.pl; 2 Department of Pharmaceutical Technology, Poznań University of Medical Sciences, 60-780 Poznań, Poland; askotnic@ump.edu.pl; 3 Department of Biophysics, Poznań University of Medical Sciences, 60-780 Poznań, Poland

**Keywords:** indapamide, differential scanning calorimetry, physical ageing, fragility, enthalpy relaxation, amorphous drugs

## Abstract

The objective of this study was to characterise amorphous indapamide (IND) subjected to the physical ageing process by differential scanning calorimetry (DSC). The amorphous indapamide was annealed at different temperatures below the glass transition, i.e., 35, 40, 45, 65, 75 and 85 °C for different lengths of time, from 30 min up to a maximum of 32 h. DSC was used to characterise both the crystalline and the freshly prepared glass and to monitor the extent of relaxation at temperatures below the glass transition (*T*_g_). No ageing occurred at 35, 40 and 45 °C at the measured lengths of times. Molecular relaxation time constants (*τ*^KWW^) for samples aged at 65, 75 and 85 °C were determined by the Kohlrausch-Williams-Watts (KWW) equation. The fragility parameter *m* (a measure of the stability below the glass transition) was determined from the *T*_g_ dependence from the cooling and heating rates, and IND was found to be relatively stable (“moderately fragile”) in the amorphous state. Temperature-modulated DSC was used to separate reversing and nonreversing processes for unaged amorphous IND. The enthalpy relaxation peak was clearly observed as a part of the nonreversing signal. Heat capacities data for unaged and physically aged IND were fitted to *C_p_* baselines of solid and liquid states of IND, were integrated and enthalpy was presented as a function of temperature.

## 1. Introduction

Active pharmaceutical ingredients (APIs) may exist in a crystalline or amorphous form. The solubility of an amorphous API can be 1.1- to 1000-fold higher in comparison to their crystalline counterpart [1,2,3,4,5,6]. Therefore, amorphous forms of drugs usually dissolve more readily and are more bioavailable than their crystalline counterparts, and for that reason, a growing interest in the development of amorphous formulations has been seen [6]. However, in contrast to crystals, amorphous materials are not thermodynamically stable [1,2,3,4,5,6,7,8,9,10,11], and the stability of the amorphous drugs is the main issue hindering its application [6]. The amorphous materials during heating do not show solid-liquid first-order phase transitions (melting), but a second-order-like solid-liquid transition called the glass transition [11]. The glass transition temperature (*T*_g_) is an important physical parameter of amorphous materials, as it indicates a border between a solid and liquid phase of low and high molecular mobility, respectively; hence, the glass transition temperature implies the conditions for the storage of amorphous API [1,6]. It has been shown that amorphous APIs may crystallise below the glass transition temperature. The process of ordering strongly depends on the storage temperature below the *T*_g_ [12]. The disordered structure of vitreous materials stored below the *T*_g_ tends to be more structurally ordered [13,14], and their total enthalpy and volume are reduced to lower values toward the equilibrium state. This phenomenon is called physical ageing [15,16,17,18,19,20,21,22,23,24,25,26]. At low temperatures, the apparent equilibrium is difficult to achieve, as the molecular mobility is low; thus, the time for observing physical ageing may be long. If the amorphous material is stored at a constant temperature lower than the *T*_g_, the phenomenon is called isothermal physical ageing. Physical ageing in a nonisothermal manner may occur when the material is cooled from a temperature above the *T*_g_ to a temperature below the glass transition temperature [16,17,18,19,20]. As a result of physical ageing, the physicochemical properties of the materials change [27,28]. Physical ageing may have an impact on the physical or chemical properties of pharmaceutical material such as, for instance, water sorption or crystallisation tendency [21,22,23,24]. In contrast to chemical or biological ageing, physical ageing is a reversible phenomenon; only ordering of the amorphous phase takes place, during which no breaking or forming of chemical bonding occurs. In the case of amorphous drugs, the process of ordering the amorphous phase may lead to, among others, reducing the solubility rate [3,4,5,29,30,31,32]. For example, for a cinnarizine-Soluplus solid dispersions system stored at 25 °C, nonlinear physical ageing was observed, and the system remained amorphous during the ageing process [33]. The ageing rate decreased over time, and constant ageing was finally reached and led to a reduced dissolution rate in the absence of crystallisation [33]. The parameter characterising the process of physical ageing is the enthalpy of relaxation, also known as recovery enthalpy (Δ*H*_r_). The evolution of the structure during the physical ageing process can be also quantified in terms of the fictive temperature (*T*_f_), first introduced by Tool [34], and recovery parameter (φ), which describes the departure of the enthalpy or volume from the equilibrium state, introduced by Kovacs [35]. Changes of these three parameters of glassy materials during physical ageing indicate that the process of structural recovery depends on thermal history and is nonexponential and nonlinear [35]. The kinetic of the physical ageing process can be described by using the Kohlrausch-Williams-Watts (KWW) equation [36,37]. Another parameter used to describe the molecular mobility of the amorphous materials, and their stability is fragility [38,39,40]. The amorphous systems are classified as either “strong” or “fragile”, based on the temperature dependence of the mean molecular relaxation time [38,39,40]. The fragility parameter (*m*) was introduced by Angell [38,39,40], and it is a parameter describing the stability of amorphous materials below the glass transition temperature [41].

**Figure 1:**
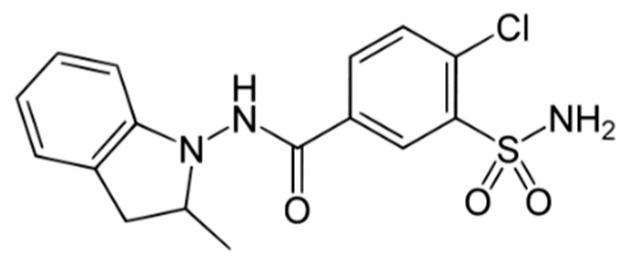
Chemical structure of indapamide.

In this study, indapamide (IND; Figure 1), a thiazide-like diuretic drug used in the treatment of hypertension and heart failure [42], was investigated. Indapamide is practically insoluble in water [43] and, as a consequence, poorly bioavailable [44]. Thus, to increase the biopharmaceutical properties of IND, it could be used in the amorphous form. Some thermodynamic properties of crystalline and amorphous aged and nonaged indapamide were published [29,30,31,45,46,47,48]. However, to our best knowledge, there is no report focusing on the comprehensive characterisation of isothermal and nonisothermal physical ageing in the context of absolute heat capacity and integral equilibrium enthalpy of the solid and liquid amorphous IND phases. The aim of this study was to investigate the physical ageing of amorphous indapamide by differential scanning calorimetry (DSC). The results are shown in the context of absolute heat capacity and total enthalpy of the solid and liquid amorphous IND phases. The fragility parameter (*m*) was determined, as well as the kinetic parameters of the isothermal physical ageing process. The Kohlrausch-Williams-Watts (KWW) equation was used to determine the relaxation time *τ*^KWW^ and the coefficient *β* describing the distribution of relaxation times during the physical ageing process.

## 2. Materials and Methods

### 2.1. Material

Indapamide (*M* = 365.835 g·mol^−1^; CAS no.: 26807-65-8) used in this study was obtained from Polpharma, Starogard Gdański, Poland. As received, indapamide was white crystalline powder in the form of hemihydrate (purity: ≥99%). Degradation of the material begins around 260 °C [47]. No degradation occurs during heating and cooling cycles in the investigated temperature range, as reported by other authors [30]. In order to obtain the amorphous form of indapamide, the samples were heated to 190 °C and subsequently cooled down to 25 °C. During the heating, water from the hemihydrate evaporated, and the crystalline phase melted, which did not recover after the sample was cooled again.

### 2.2. Standard DSC Measurements and Isothermal Physical Ageing

DSC curves were obtained using differential scanning calorimeters DSC2920, DSC Q1000 and DSC Q2500 from TA Instruments (New Castle, DE, USA) under the nitrogen gas flow 50 mL·min^−1^. The instrument calibration was performed by carrying out the measurements for reference materials with known parameters, such as the melting point, the heat of fusion and specific heat capacity. The standard for calibration of the melting point and heat of fusion was indium (*T*_m_ = 156.6 °C, Δ_f_*H* = 28.45 J·g^−1^). Sapphire (Al_2_O_3_) was used to calibrate the specific heat capacity.

Samples of IND of 5−10 mg were tightly packed into standard aluminium crucibles with pierced lids. An empty aluminium pan, identical and with the similar weight to that used for the sample, was used as the reference. For the standard DSC measurements, the samples were equilibrated at 25 °C for 5 min and then linearly heated with a heating rate of 10 °C·min^−1^ to 190 °C and held for 5 min to melt the crystalline form. Next, the samples were cooled to 25 °C and heated again to 190 °C at 10 °C·min^−1^ rate. This heating-cooling cycle was repeated.

The isothermal process of physical ageing of amorphous indapamide was carried out at ageing temperatures 35, 40, 45, 65, 75 and 85 °C. For the annealing cycle, the molten IND was subjected to the following process: cooled at 10 °C·min^−1^ to the ageing temperature, held at this temperature for a particular ageing time, then cooled further to 25 °C at 10 °C·min^−1^, held for 5 min and heated at 10 °C·min^−1^ to 190 °C. All of the measurements were performed at least in two repeats, and the results presented in this paper are the average values. The DSC data were analysed using TA Universal Analysis 2000 (v. 4.5A, TA Instruments—Waters LLC, New Castle, DE, USA).

### 2.3. Temperature-Modulated Differential Scanning Calorimetry (TMDSC) Measurement

The TMDSC measurements of indapamide consisted of heating the sample with the underlying heating rate of 1 °C·min^−1^ and a temperature modulation with an amplitude of 1 °C and a period of 60 s. The measurements were performed within the temperature range 25−190 °C.

### 2.4. Determination of Fragility Parameter

The fragility parameter was determined from the *T*_g_ or *T*_f_ dependence from various heating and cooling rates [5,49,50]. In the experiment with different heating rates, cooling with a constant rate (20 °C·min^−1^) was followed by different heating rates (0.5–20 °C·min^−1^). For nonisothermal ageing, different cooling rates (0.2–10 °C·min^−1^) were applied, and then, the sample was heated with a constant heating rate of 10 °C·min^−1^. The measurements were performed within the temperature range 25−190 °C. At 25 °C, the sample was held for 5 min and, at 190 °C, for 3 min.

## 3. Results and Discussion

**Figure 2:**
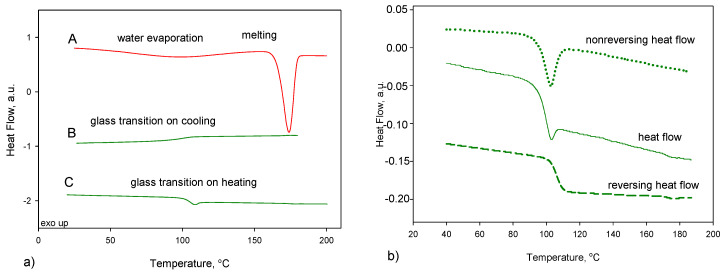
The experimental (**a**) differential scanning calorimetry (DSC) data of the heat-flow rate vs. temperature for indapamide. A—1st heating run for hemihydrate crystalline material (red), B—cooling run from the melt (green), C—heating run of amorphous indapamide (IND) (green) and (**b**) temperature-modulated differential scanning calorimetry (TMDSC) data of the heat-flow rate vs. temperature for the heating run of amorphous IND. Total heat-flow curve (green solid)—showing the glass transition process overlapped with the enthalpy relaxation peak, reversing the heat-flow curve (green dashed)—showing the glass transition process only and nonreversing the heat-flow curve (green dotted)—showing the enthalpy relaxation peak only.

Figure 2a shows the standard DSC trace of crystalline indapamide (hemihydrate) on heating (curve A) and melted IND on cooling (curve B) and consecutive reheating runs (curve C). The glass transition phenomenon is observed on the cooling and reheating runs and confirms the full amorphisation of the IND. The glass transition temperature, *T*_g_, value during cooling (curve B on Figure 2a) was estimated as 97.9 ± 0.6 °C, which is in good agreement with the literature, i.e., 98 °C [48] and 102.2 ± 1.1 °C for heating runs similar to previously reported data: 103.85 °C [29], 104.7 °C [30] and 102 °C [31]. Change in the specific heat capacity during the glass transition process is equal to 0.43 J·g^−1^·°C^−1^ and 0.47 J·g^−1^·°C^−1^ for cooling and heating the sample, respectively. On the curve obtained during heating of the amorphous sample (Figure 2a, curve C), a small peak corresponding to the enthalpy relaxation is observed, arising from an additional ordering of the amorphous phase during the measurement due to the nonisothermal ageing below the *T*_g_. A similar experiment was already published by us [47], but for the clarity of this work, it is shown again.

To observe the glass transition (thermodynamic phenomenon) not affected by the enthalpy relaxation (kinetic phenomenon) associated with the physical ageing occurring during the standard DSC measurement, the TMDSC measurement was performed. The deconvolution of the total heat flow signal into the reversing and nonreversing heat flows can be obtained by using a modulated mode [51,52]. Figure 2b presents the total heat-flow signal separated into reversing and nonreversing heat flows for amorphous indapamide. On the reversing heat flow, only thermodynamic phenomenon can be seen, and, in this case, it is the glass transition not affected by the physical ageing process. The glass transition temperature obtained from reversing heat-flow data was estimated as 106.1 °C, and the change of heat capacity, Δ*C_p_*, at this temperature has a value of 0.36 J·g^−1^·°C^−1^. Nonreversing heat-flow data gives information about the kinetic phenomenon, and thus, only the peak of the enthalpy relaxation is observed.

The fragility parameter (*m*) was determined from the *T*_g_ and *T*_f_ dependence on the heating and cooling rates, respectively. The fragility is used to describe the non-Arrhenius temperature dependence of the structural relaxation time and is best quantified using the fragility parameter as the effective activation enthalpy scaled by the available thermal energy at the *T*_g_ [53,54]. The fragility parameter is of great interest for the pharma industry, as it can be used to predict the tendency of amorphous drugs toward recrystallisation [5]. Kawakami et al. correlated a glass-forming ability (GFA—describes properties related to crystallisation behaviours) with fragility [41,50,55]. The amorphous materials characterised by a low value of *m* are classified as “strong” glasses, whereas a large value of *m* as “fragile” glasses. “Strong” glasses are expected to be more physically stable than fragile materials [5,55]. The correlation found by Kawakami et al. [50] suggests that “fragile glass” has a low GFA. In the case of amorphous APIs, the typical *m* values range between 60 and 120 [5,50,56]. The glasses with higher *m* parameters have stronger frustrations against crystallisation. This frustration of the materials results from the competition between long and short ordering occurring during cooling from a liquid to a solid state. Long-range ordering is responsible for nucleation and crystal growth, while short-range ordering is associated with the formation of the local structures that have no crystallographic symmetry [55]. The value of the kinetic fragility (*m*) and the energy activation (*E*_a_) of the glass transition of IND were determined from the plots based on the following equations:

(1) $$m=\frac{E_{a}}{2.303\cdot \;R\cdot T_{g}},$$

(2) $$E_{a}=\frac{\mathrm{dln}\left(q\right)}{d\left(T_{g}{}^{-1}\right)},$$

where *R* is the gas constant, and *q* is the heating or cooling rate.

**Figure 3:**
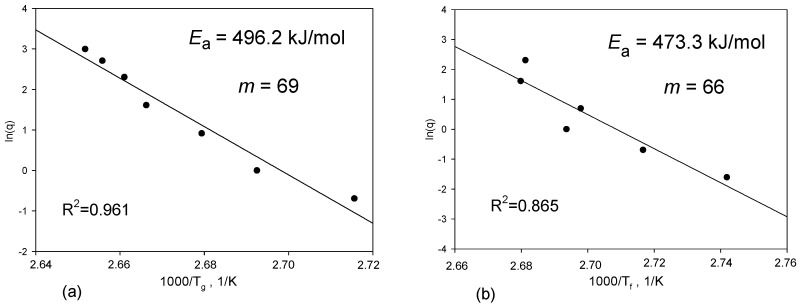
Energy activation (*E*_a_) and fragility parameter (*m*) for indapamide obtained from the dependence of the glass transition temperature (*T*_g_) and fictive temperature (*T*_f_) on (**a**) the heating and (**b**) cooling rates.

Figure 3 presents the dependence of the glass transition temperature and fictive temperature on heating/cooling rates together with the linear regression line. The slope gives the apparent activation energy, *E*_a_. Good linearity is observed between the *T*_g_ and heating/cooling rates. The heating and cooling modes lead to similar results of the fragility parameter, i.e., *m*_heating_ = 69 and *m*_cooling_ = 66. Moreover, the obtained values are in reasonable agreement with the values reported by the group of Paluch, i.e., *m* = 76 [30] and *m* = 73 [46]. Based on the classification system proposed by Angell [57,58,59], IND can be classified as “moderately fragile” glass.

The amorphous materials stored below the *T*_g_ undergo the physical ageing process, showing a gradual loss in energy in terms of enthalpy due to the effect of ordering and the limitation of molecular motions occurring below the *T*_g_ [27,60,61,62,63]. The amount of enthalpy lost during storage below the *T*_g_ is recovered by the sample during its heating run in DSC. The recovery enthalpy can be measured with the ageing time, and it reflects the ordering, loss of the molecular mobility and, thus, the physical stability. The process of physical ageing of amorphous indapamide carried out at the ageing temperature range from 30 to 45 °C showed that no ageing occurs in the investigated time frame (Figure S1, Supplementary Materials). However, during the annealing of indapamide at temperatures closer to the glass transition temperature (*T*_a_ = 65, 75 and 85 °C) changes in the heat capacity originated from the physical ageing process can be observed.

**Figure 4:**
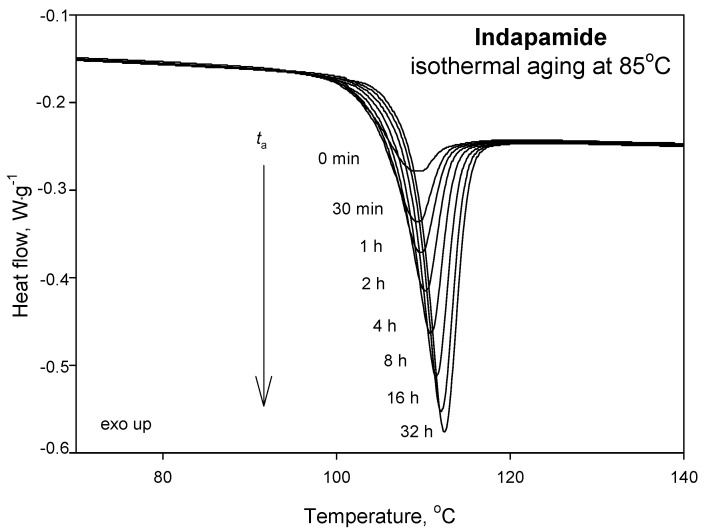
Heat-flow plot as a function of the temperature obtained from DSC measurements during heating indapamide with a constant heating rate of 10 °C·min^−1^ after isothermal ageing at 85 °C for various ageing times, up to 32 h.

Figure 4 presents the heat-flow as a function of the temperature in the glass transition region obtained from DSC measurements of amorphous IND aged isothermally at 85 °C for various ageing times, from 30 min up to 32 h. It can be seen that, as the ageing time increases, the endothermic peak of the enthalpy relaxation increases and moves toward higher temperatures. Serajuddin et al. [48] presented the process of isothermal physical ageing of indapamide at 85 °C, and it was found that, after 168 h, the enthalpy relaxation reaches its maximum value; however, the values were not provided.

**Figure 5:**
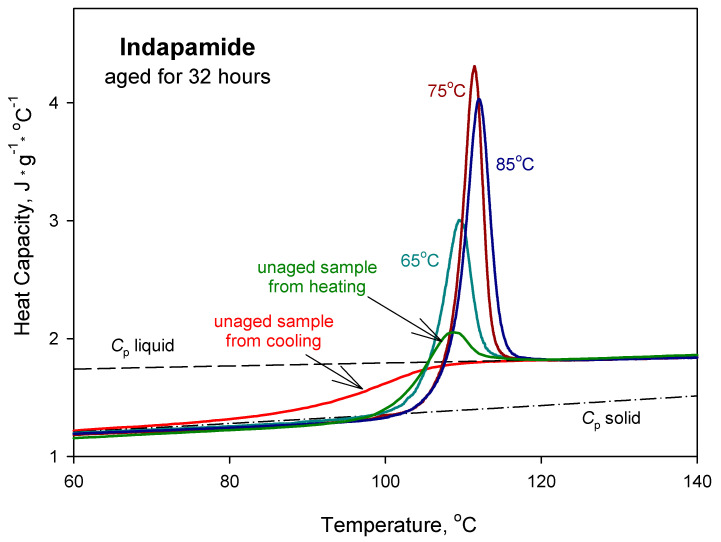
Experimental heat capacity as a function of temperature for amorphous IND aged isothermally at 65, 75 and 85 °C for 32 h, together with the solid heat capacity *C*_p_(solid) and liquid heat capacity *C*_p_(liquid) as reference lines [47]. *C*_p_ of the unaged sample was obtained during cooling (red line) from the molten state and heating (green line) after cooling from the melt. Scans were recorded with a constant heating/cooling rate of 10 °C·min^−1^.

Figure 5 presents the comparison of the experimental heat capacity results obtained for the ageing of IND for 32 h at 65, 75 and 85 °C. Data are fitted to the solid *C*_p_(solid) and liquid C_p_(liquid) heat capacity baselines for IND published earlier [47]. The heat capacity for unaged IND was obtained during cooling with a constant cooling rate of 10 °C·min^−1^ from the molten state and during heating the sample with a constant rate of 10 °C·min^−1^ after cooling it from the melt. On heating of the unaged sample, a small enthalpy relaxation peak occurs as a result of ordering the molecules during measurements. The value of enthalpy relaxation for ageing at 75 °C is higher than for 85 °C. This may be due to that, during cooling, the vitrification process starts at lower temperatures, and 85 °C may be in the region of this broad glass transition region.

**Figure 6:**
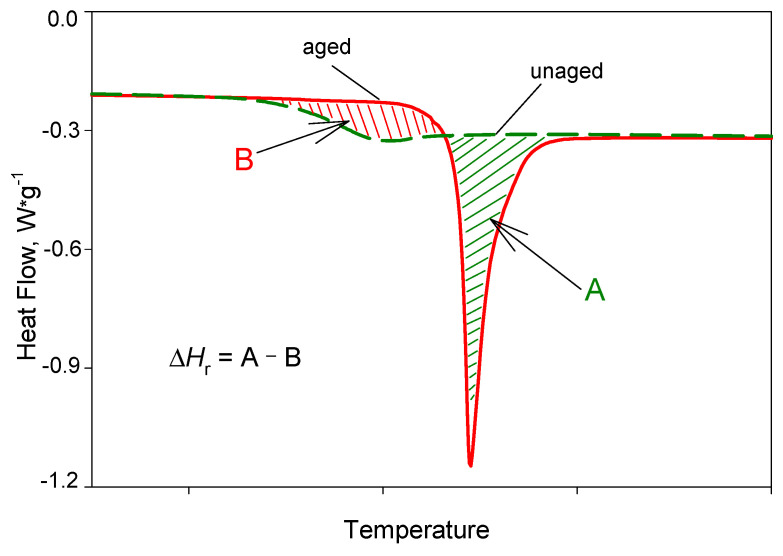
Scheme for the determination of the enthalpy relaxation (Δ*H*_r_) from the plots of heat-flow vs. temperature. The enthalpy relaxation was calculated by subtracting the areas created between aged and unaged samples.

Enthalpy relaxation was calculated from the difference of the areas created between the heat flow of the aged and unaged samples (Δ*H*_r_ = A − B) on the heat-flow vs. temperature plots (see Figure 6), which, mathematically, can be represented by the equation:

(3) $$\Delta H_{r}=\frac{1}{q}\cdot \int_{T1}^{T2}\left(\phi_{aged}-\phi_{unaged}\right)dT=\int_{T1}^{T2}\left(C_{p\mathrm{aged}}-C_{p\mathrm{unaged}}\right)dT,$$

where *q* is the heating rate, *T*_1_ and *T*_2_ are limits of the integration (where *T*_1_ < *T*_g_ < *T*_2_); *Φ*_aged_ and *Φ*_unaged_ are heat flows originating from the aged and unaged samples, respectively, *C*_p__aged_ and *C*_p__unaged_ are the heat capacities of the aged and unaged samples, respectively, and *T* is the temperature. The areas were determined using TA Universal Analysis 2000 software by integrating the obtained heat-flow vs. temperature data within the appropriate temperature limits.

**Figure 7:**
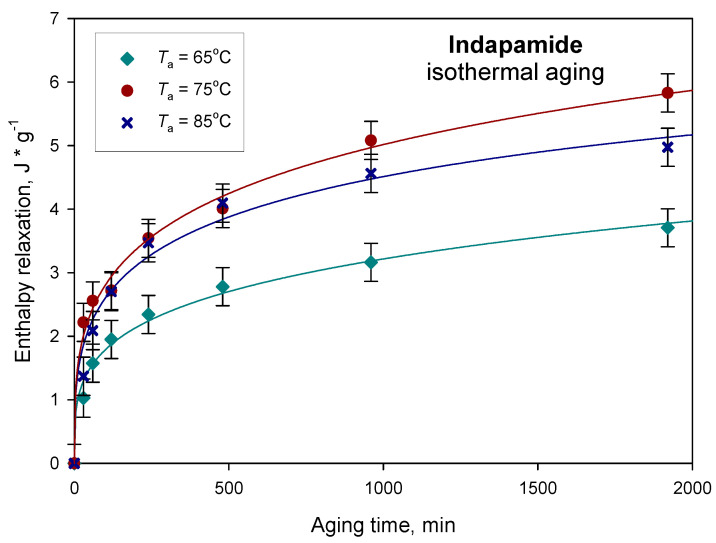
Enthalpy relaxation as a function of the ageing time for indapamide aged at 65, 75 and 85 °C.

The calculated enthalpy relaxation as a function of the ageing time for amorphous IND aged at 65, 75 and 85 °C are shown in Figure 7. Data were presented as the average values with the error bars for the standard deviation (±0.3 J·g^−1^). Initially, the changes in the enthalpy relaxation are noticeable, and after about 10 h, the differences between enthalpies for the subsequent ageing times decrease, and the value of enthalpy approaches the equilibrium enthalpy relaxation.

Next, the enthalpy relaxation for an infinite ageing time, labelled as the equilibrium enthalpy relaxation, $\Delta H_{r}^{\inf}$, was estimated from the following equation:

(4) $$\Delta H_{r}^{\inf}=\int_{Ta}^{Tg}\Delta C_{p}dT\;\approx \Delta C_{p}\left(T_{g}-T_{a}\right),$$

where Δ*C*_p_ is the change of the heat capacity of solid and liquid states in a given temperature, and *T*_g_ and *T*_a_ are the glass transition and ageing temperature, respectively.

There are several empirical models describing the kinetics of the physical ageing process [34,35,36,37,64,65,66,67,68,69]. One of such models is the Kohlrausch-Williams-Watts (KWW) approach [35,36], defined by the equation:

(5) $$\Delta H_{r}\;=\;\Delta H_{r}^{\inf}\;[1-\exp\{-{\left(t/\;\tau^{\mathrm{KWW}}\right)}^{\beta }\}],$$

or, after modification:

(6) $$\varphi =1-\frac{\Delta H_{r}}{\Delta H_{r}^{\inf}}\;=\;\varphi \left(t\right)=\exp\left\{-{\left(t/\;\tau^{\mathrm{KWW}}\right)}^{\beta }\right\},$$

where Δ*H*_r_ and $\Delta H_{r}^{\inf}$ are the enthalpy of relaxation and equilibrium enthalpy relaxation, respectively, *t* is the ageing time, *τ*^KWW^ is the relaxation time and *β* is the coefficient describing the distribution of the relaxation time. When *β* is small, the distribution of the states is wide. The modification of KWW Equation (5) leads to another form of this, Equation (6), and another quantity, a recovery parameter (φ).

The experimental data of Δ*H*_r_ were fitted to the KWW Equation (5) as a function of time *t*. From the best fit, the KWW parameters *τ*^KWW^ and *β* were obtained to describe the kinetics of the physical ageing of amorphous indapamide under tested conditions. In Figure 7, the calculated enthalpy relaxations according to the Equation (5) are presented as solid lines for different ageing temperatures (65, 75 and 85 °C). Calculated values of the relaxation time *τ*^KWW^ and coefficient *β* are shown in Table 1. As the storage temperature increased and approached the *T*_g_, a significant drop in *τ*^KWW^ was observed, from days to hours, i.e., 189 days (65 °C) and 6 days (75 °C) to 28 h (85 °C). The distribution of the relaxation times slightly increased with an increase in the annealing temperature.

**Figure 8:**
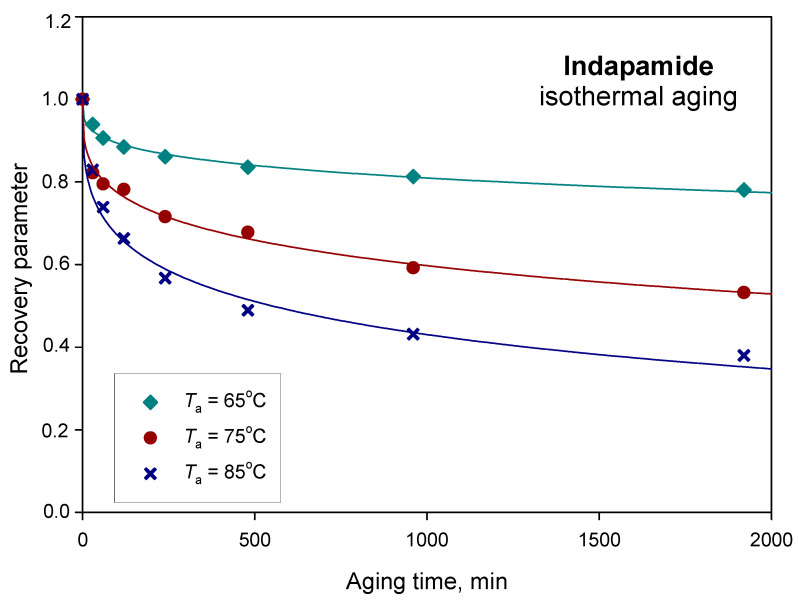
Recovery parameter as a function of the ageing time for amorphous indapamide aged at 65, 75 and 85 °C.

The recovery parameter (φ) directly describes the kinetics of physical ageing and the extent relaxation of the glassy material with the ageing time. The relationship in Equation (6) was used for the estimation of the experimental φ values for different ageing times. Both the experimental (dots) and calculated (solid lines) recovery parameters are presented in Figure 8.

The enthalpy relaxation changes caused by physical ageing are the highest for the process carried out at 75 °C, as can be seen in Figure 7; however, the relationship between the recovery parameter and the ageing time for the performed tests shows that the physical ageing process of indapamide is the fastest at ageing temperature *T*_a_ = 85 °C, which can be seen in Figure 8. The higher the value of φ, the lower the relaxation of glass toward the equilibrium supercooled state. It was observed that the recovery parameter φ of IND after annealing for 32 h at ageing temperatures 65, 75 and 85 °C changed from 0.78 and 0.53 to 0.38, respectively. A similar tendency was observed for different pharmaceutical materials. For example, the amorphous irbesartan molecular relaxation time constant decreased from 302 years to 68 h with the increase in annealing temperature from 25 to 40 °C [70]. Additionally, the *β* parameter increased with the ageing temperature from 0.19 for *T*_a_ = 25 °C to 0.37 for *T*_a_ = 40 °C. In other studies, a comparison of the relaxation times for sucrose glass quenched from the melt also showed a decrease in the relaxation time with ageing temperatures closer to the *T*_g_ [71]. It should be noted that the relaxation time value for any given ageing condition can vary depending on the sample preparation. Chieng et al. [72] reported that the relaxation times for the samples prepared by freeze-drying have significantly shorter relaxation times than samples prepared by quenching from the melt.

**Figure 9:**
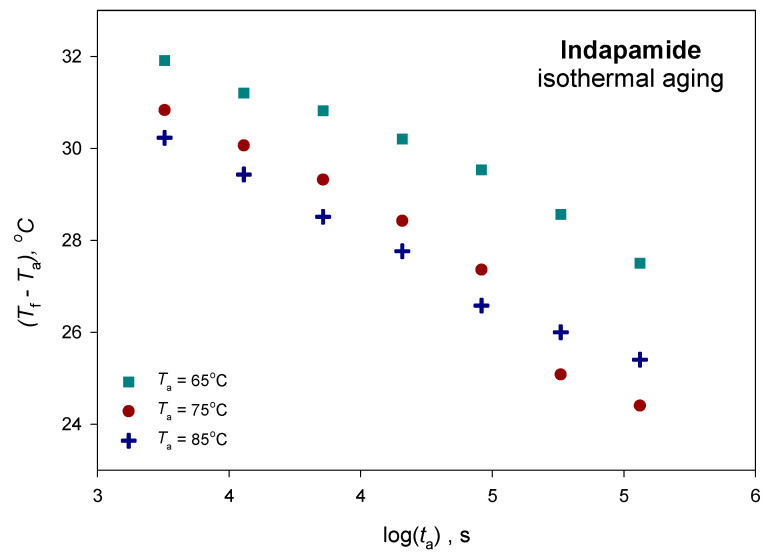
The changes of fictive temperature (*T*_f_) with annealing time (*t*_a_) for the studied aging temperatures (*T*_a_).

For all of the aged IND samples, the values of the fictive temperature, *T*_f_, has been determined by estimation of the intersection of the extended line of enthalpy of the liquid state with the enthalpy of the solid state of the aged sample, and they are presented in the Supplementary Materials (Table S1). Figure 9 presents the changes of the fictive temperature with the aging time. This presentation has the advantage of expressing the fictive temperature with the temperature of the annealed glass relative to the annealing temperature, which also represents the theoretical equilibrium fictive temperature. The value of the fictive temperature decreases with the increasing aging time. For the unaged sample, the fictive temperature is equal to the glass transition temperature, and with the aging time, it decreases to the aging temperature value for the sample where enthalpy relaxation reached the value of equilibrium enthalpy relaxation [73].

**Figure 10:**
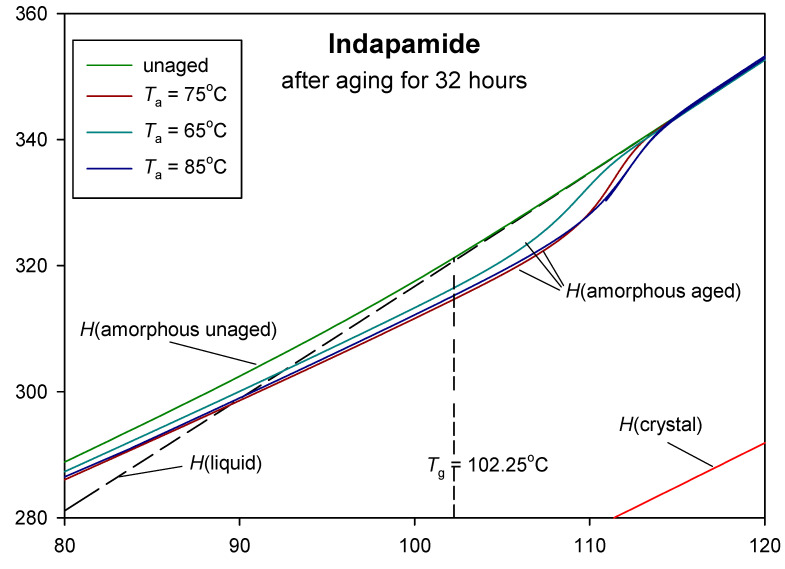
Changes of the total enthalpy vs. temperature for amorphous indapamide aged (*H*(aged)) and unaged (*H*(unaged)) in the glass transition region; dashed line is the extrapolation of the enthalpy of the liquid state (*H*(liquid)), and *H*(crystal) is the total enthalpy of the crystalline IND sample.

Figure 10 presents the total enthalpy graph as a function of the temperature of aged and unaged indapamide at the different ageing temperatures at 65, 75 and 85 °C. The total enthalpy of the unaged amorphous indapamide was determined by integrating the specific heat capacity obtained during cooling from the molten state to the amorphous solid state [47] and for the aged sample of IND by integrating the specific heat capacity obtained during heating after cooling from melt to the ageing temperature and storing this material for 32 h.

In Figure 10 the equilibrium enthalpies of the solid crystal and liquid were designated as *H*(crystal) and *H*(liquid), respectively, and were used as reference lines in the advanced thermal analysis of indapamide. All the integral enthalpies in Figure 10 were calculated for all the data presented in Figure 5. The dashed line in Figure 10 presents the extrapolation of enthalpy of the liquid state to a temperature far below the *T*_g_. The integral enthalpy of unaged amorphous IND is characterised by the glass transition temperature *T*_g_ = 102.3 °C (on curve *H*(amorphous aged)), where the function of enthalpy changes the slope. Based on the intersection of the dashed line *H*(liquid) and experimental enthalpy of aged *H* (amorphous aged), the fictive temperature *T*_f_ was determined for ageing for 32 h at 65, 75 and 85 °C, giving the results, respectively, of 92.5, 89.4 and 90.4 °C. For the aged samples, the enthalpy relaxation was estimated as the difference between the total enthalpy of the unaged and aged samples at the given ageing temperature. The difference between the total enthalpy value of the unaged sample and the value on the extended enthalpy line of the liquid state at the ageing temperature is the equilibrium enthalpy of relaxation ($\Delta H_{r}^{\inf}$).

Both the enthalpy relaxation and equilibrium enthalpy relaxation values obtained from the total enthalpy plot differ from those previously calculated. The enthalpy relaxations for ageing for 32 h at 65, 75 and 85 °C determined from Figure 10 are, respectively, 1.3, 2.5 and 3.0 J·g^−1^. The equilibrium enthalpy relaxations are more similar to those previously calculated: 15.7 J·g^−1^ for the aging at 65 °C, 10.2 J·g^−1^ for 75 °C and 5.5 J·g^−1^ for 85 °C.

It should be noted that the analysis of the isothermal physical ageing of IND at 85 °C for 32 h in the presentation of integral enthalpy was already presented in reference [47]. In this paper, the research was extended to other annealing temperatures and ageing times, and the details are shown in Figure 10 in the glass transition region from 80 to 120 °C (353 to 393 K). In the Supplementary Materials, the already published plot [47] of integral enthalpies vs. temperature for crystalline, unaged amorphous and aged amorphous IND at 85 °C for 32 h in the broader region of temperatures from −73 to 227 °C (200 to 500 K) showing all phase transitions is presented for a better understanding of the system (Figure S2).

## 4. Conclusions

The kinetics of the isothermal physical ageing process of amorphous indapamide at different ageing temperatures (65, 75 and 85 °C) were conducted. No ageing occurred at 30, 40 and 45 °C at the analysed time range. At higher temperatures, the ageing process was observed and increased when approaching the glass transition temperature. The maximum enthalpy relaxation occurred at *T*_a_ = 75 °C. The enthalpy recovery (relaxation) and the fictive temperature for IND were determined. The results of the enthalpy relaxation were fitted to the KWW equation, and the parameters describing the kinetics of the ageing process of amorphous IND, such as the relaxation time *τ*^KWW^ and *β* parameter, were obtained. The physical ageing of IND underwent the fastest at 85 °C. Additionally, the nonisothermal physical ageing process was performed, and the results were used to calculate the fragility parameter of the drug. According to the fragility parameter, IND can be classified as “moderately fragile”. The study clearly showed that IND is a stable glass in the analysed temperatures and ageing times and can be potentially used in the amorphous state in the formulations. The obtained results of the experimental heat capacity were calibrated to the baselines of *C*_p_(solid) and *C*_p_(liquid) as reference lines. The heat capacities were integrated to obtain the total enthalpy as a function of the temperatures for unaged and aged samples of amorphous indapamide. The knowledge of the detailed thermodynamic and kinetic parameters of amorphous IND allows obtaining a comprehensive characterisation of the material, which is of great importance to understand and predict its macroscopic physiochemical properties, such as stability, which is of paramount importance for the final dosage form.

## Figures and Tables

**Table 1:** Parameters describing the kinetics of the physical ageing process of indapamide at ageing temperatures (*T*_a_) 65, 75 and 85 °C determined by the experimental data fitted to the Kohlrausch-Williams-Watts (KWW) equation. $\Delta H_{r}^{\inf}$ is the equilibrium enthalpy relaxation, *τ*^KWW^ is the relaxation time and *β* is the coefficient describing the distribution of the relaxation time.

Parameters	*T*_a_ = 65 °C	*T*_a_ = 75 °C	*T*_a_ = 85 °C
$\Delta H_{r}^{\inf}$ (J·g^−1^)	16.88	12.45	7.92
β	0.277	0.306	0.327
$\;\tau^{\mathrm{KWW}}$	~189 days	6 days	28 h

## Supplementary Materials

The following are available online at https://www.mdpi.com/1999-4923/12/9/800/s1: Figure S1: Heat-flow vs. temperature plots from the physical ageing of indapamide at (**a**) 35, (**b**) 40 and (**c**) 45 °C. Figure S2: Experimental enthalpy of crystalline *h*^DSC^(exp-cr) and amorphous unaged *h*^DSC^(exp-am on cooling) and aged *h*^DSC^(exp-aged) indapamide as measured by DSC in the frame of the calculated enthalpy in the solid and *h*(crystal) = *h*(solid) and liquid and *h*(liquid) states. Additionally, the enthalpy of aged amorphous indapamide, *h*^DSC^(exp-aged), is presented (*T*_a_ = 85 °C for 32 h at 0% relative humidity (RH)). Table S1: Fictive temperature (Tf) vs. aging time (ta) of indapamide aged at Ta = 65, 75 and 85 °C.
