# Supplementary material for: Physical Ageing of Amorphous Indapamide Characterised by Differential Scanning Calorimetry
Source: Pharmaceutics. 2020 Aug 25;12(9):800. doi: 10.3390/pharmaceutics12090800 (PMC7558952; doi:10.3390/pharmaceutics12090800)
Supplement: Supplementary file 1 [file pharmaceutics-12-00800-s001.pdf]

pharmaceutics

MDPI

Supplementary Materials: Physical Ageing of Amorphous Indapamide Characterized by Differential Scanning Calorimetry

Agata Drogoń, Marcin Skotnicki, Agnieszka Skotnicka and Marek Pyda

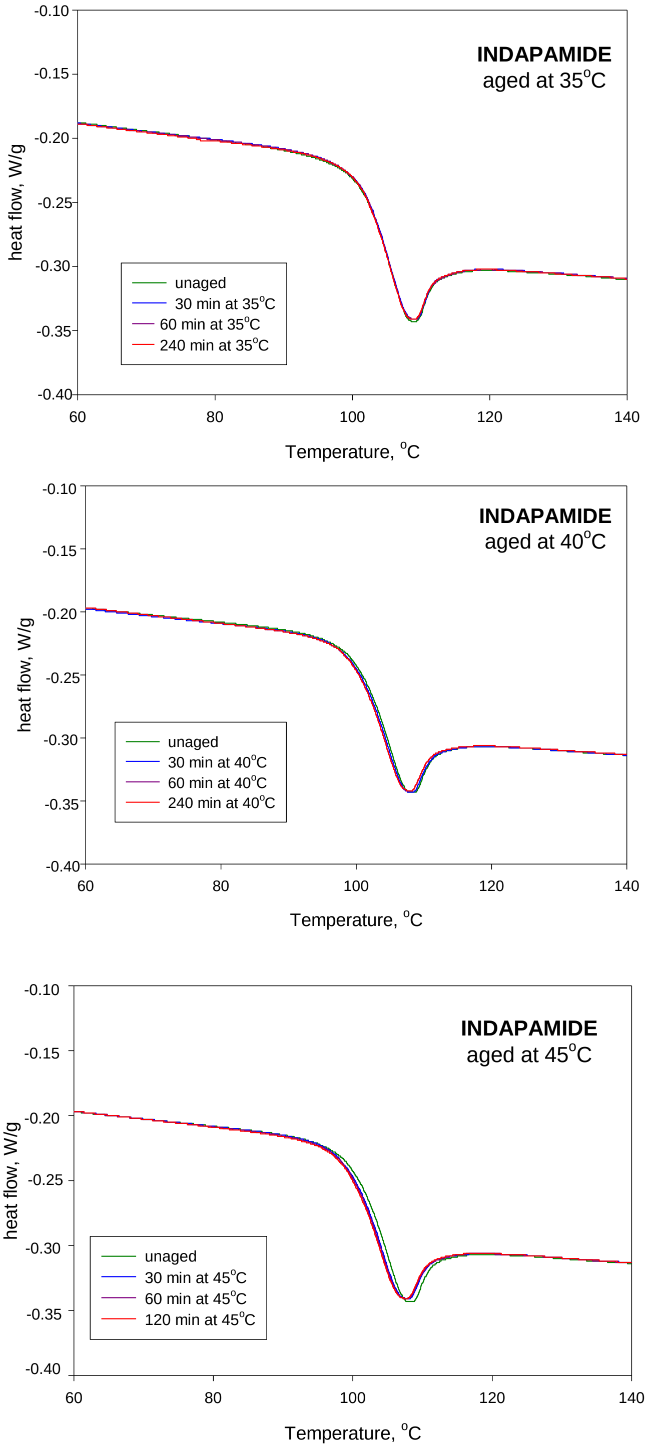

Figure S1. Heat-flow vs temperature plot from physical ageing of indapamide at (a) 35, (b) 40 and (c) 45°C.

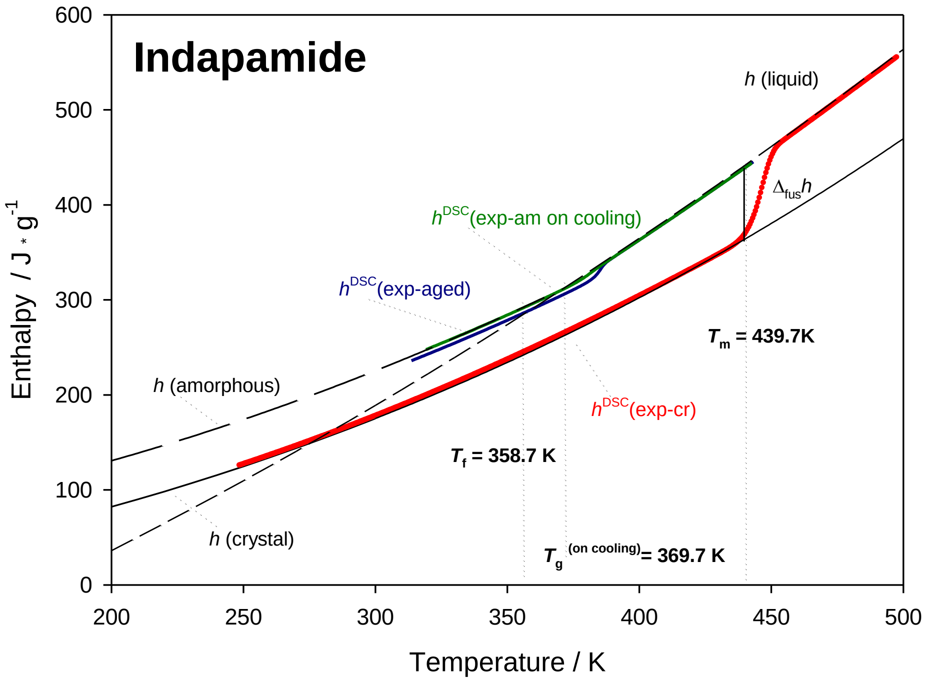

Figure S2. Experimental, enthalpy of crystalline, h^{DSC}(exp-cr) and amorphous, unaged h^{DSC}(exp-am on cooling), aged, h^{DSC}(exp-aged), indapamide as measured by DSC in the frame of calculated enthalpy in the solid, *h*(crystal) = *h*(solid) and liquid, *h*(liquid) states. Also, the enthalpy of aged amorphous indapamide, h^{DSC}(exp-aged), is presented (T_a = 85°C for 32 hours at 0% RH). Reprinted from Skotnicki *et al.*, Thermochim. Acta. 674, 36–43, 2019, with permission from Elsevier [1].

Table S1. Fictive temperature (T_f) vs. aging time (t_a) of indapamide aged at T_a = 65, 75 and 85 °C.

**Annealing time, t_a (min)**	**Fictive Temperature T_f (°C)**		
	**T_a = 65°C**	**T_a = 75°C**	**T_a = 85°C**
30	96.91	95.83	95.23
60	96.20	95.06	94.43
120	95.82	94.32	93.51
240	95.20	93.42	92.76
480	94.53	92.36	91.58
960	93.56	90.08	91.00
1920	92.50	89.40	90.40

References

1. Skotnicki, M., Drogoń, A., Calvin, J., Rosen, P., Woodfield, B., Pyda, M., 2019. Heat capacity and enthalpy of indapamide. Thermochim. Acta 674, 36–43.
